# Changes in the Quality of *Idesia polycarpa* Maxim Fruits from Different Ecotypes During the Growth Process

**DOI:** 10.3390/plants14152324

**Published:** 2025-07-27

**Authors:** Yi Yang, Chao Miao, Qiupeng Yuan, Wenwen Zhong, Zuwei Hu, Chen Chen, Zhen Liu, Yanmei Wang, Xiaodong Geng, Qifei Cai, Li Dai, Juan Wang, Yongyu Ren, Fangming Liu, Haifei Lu, Tailin Zhong, Zhi Li

**Affiliations:** 1 College of Forestry, Henan Agricultural University, Zhengzhou 450046, China; 2 National Forestry and Grassland Administration Key Laboratory for Central Plains Forest Resources Cultivation, Zhengzhou 450046, China; 3 Henan Province Engineering Technology Research Center for *Idesia*, Zhengzhou 450046, China; 4 College of Urban Construction, Zhejiang Shuren University, Shaoxing 312028, China

**Keywords:** *Idesia polycarpa*, fruit, quality, yield, seed source variation

## Abstract

The goal of this study was to build an understanding of the quality of *Idesia polycarpa* fruit Maxim from different ecotypes and to identify the best cultivars, with a view to providing a reference and theoretical basis for the selection and cultivation of *I. polycarpa*. In this study, we systematically evaluated the fruit quality characteristics of five seed sources, namely SH, SG1, GG, HX, and SG2, at four developmental stages, M1-M4, through a principal component analysis, a correlation analysis, and a significance test. Comparisons between the ecotype yielded that GG was significantly better than the other ecotype in oil content (28.7%) and fresh weight per cluster (155.56 g), while HX exhibited higher SOD content (278.18 U/g) and soluble protein content (27.50 mg·g^−1^), suggesting a higher level of stress tolerance. The results of the correlation analysis showed that POD was significantly negatively correlated with oil content (r = −0.633) and SOD (r = −0.617) activities, indicating that the antioxidant enzyme system may affect oil accumulation. The results of the principal component analysis showed that the cumulative contribution of the first four principal components reached 89.72%, of which principal component 1 mainly reflected yield-related traits, and principal component 2 was significantly correlated with oil content and soluble protein. Through the evaluation and screening of the five ecotypes, we determined that GG can be utilized as a good single plant in the selection and improvement of new cultivars; our findings can provide theoretical support for the selection of good cultivars of *I. polycarpa* seed in the central region of Henan.

## 1. Introduction

*Idesia polycarpa* Maxim belongs to Salicaceae; the plant is also known as oil grapes, water winter melon, mountain sycamore, etc., and is referred to as the “tree on the oil bank” [1]. China is a center for *I. polycarpa* distribution. Most provinces in China have a distribution of wild *I. polycarpa*; its wide distribution and adaptability mean that it is an excellent tree species for use in the afforestation and greening of deserted mountains [2]. The tree crown of *I. polycarpa* is of a layered type; the fruit is bright red in color after maturity, with a string-hanging sequence; the wood hardness is moderate; this species is adaptable, and can be used as an ornamental, ecological wood species in gardening and greening applications [3]. The fruit yield of *I. polycarpa* is very high, measuring more than 1.5 kg/m^2^ [4]. The fruit of *I. polycarpa* can be used as a raw material in various applications: edible oil extraction [5]; linoleic acid extraction [6,7]; bio-lubricant production [8,9]; other aspects of preparation technologies. Developments in the *I. polycarpa* industry have laid a solid national foundation. The pulp oil content from the plant can be as high as 44.08% [10], with a seed oil content of 26.54%; accordingly, *I. polycarpa* is an important woody oilseed species in China [11].

Fruit morphologies and physiological and biochemical indices are fundamental parameters that must be determined in elucidating the mechanisms underlying fruit growth and development. In recent years, several studies have explored various aspects of *I. polycarpa*. For example, the oil content and fatty acid composition of *I. polycarpa* from five regions were analyzed by Wang et al. [12]. Their findings indicated that fruits from Henan Province exhibited the highest oil content, while oil derived from *I. polycarpa* had the highest linoleic acid content. Seedling growth indices of *I. polycarpa* of 12 provenances were measured by Jiang et al. [13], who determined that seedlings of the Fanyi provenance showed the fastest growth rate. According to Gong et al. [14], who investigated the fruit fatty acid profiles of *I. polycarpa* samples from 11 provenances, the fruits are rich in unsaturated fatty acids, with linoleic acid being the predominant component. Hernández et al. [15] showed that the fatty acid profile (especially oleic/linoleic acid ratio) and its genomic expression have a significant effect on oil stability. The chemical composition and antioxidant activity of the pulp oil from five places in China were analyzed by Zhang et al. [16]; meanwhile, Ye et al. [17] optimized the extraction process of polyphenols from the defatted fruit residue of S. sanguinis and their in vitro/in vivo antioxidant and whitening activities. The photosynthetic characteristics of *I. polycarpa* samples from four provenances were investigated by Xu et al. [18]; their results showed that the sample of Zhangjiajie provenance had the highest light adaptability, while that of Tokyo provenance had the widest range of net photosynthetic rate (Pn) and the highest Pn value. A quality evaluation of seven provenances of *I. polycarpa* fruits and their oils was carried out by Zou et al. [19], who found provenance to be a significant factor influencing quality. Experiments on *I. polycarpa* under different environmental conditions were performed by Xu et al. [20], with the results demonstrating strong resistance characteristics. The response mechanism of *I. polycarpa* under drought stress [21] and flood stress [22] was explored by Geng et al., revealing that moderate drought could promote seedling growth while showing some tolerance to flood stress. Research conducted by Tian et al. [23] on *I. polycarpa* under extreme high temperatures and high temperature–drought stress showed that high temperature–drought stress caused greater damage than high-temperature stress alone. In addition, significant progress has been made in research on the physiological mechanisms and cultivation management of *I. polycarpa*, an ecologically and economically valuable tree species. Feng et al. addressed disease issues in practical production by systematically identifying the pathogen responsible for stem canker in *I. polycarpa* for the first time and developing effective chemical control strategies, providing a scientific basis for integrated disease management [24]. In the field of root ecology, Li et al. evaluated the fine root morphology and rhizosphere environmental characteristics of dioecious *I. polycarpa*, revealing the potential influence of gender differentiation on root architecture and soil microenvironments, thereby laying the foundation for sex-specific cultivation practices [25]. Zhang et al. focused on the regulatory mechanisms of seed germination. Through transcriptome analysis, they elucidated the molecular network by which temperature drives seed germination through the coordinated regulation of plant hormone signaling and sugar metabolism pathways [26]. Regarding nutrient management, Wang et al. investigated the effects of varied phosphorus fertilizer ratios on the rhizosphere microbial community structure in seedlings. Their study confirmed that phosphorus levels significantly alter bacterial and fungal diversity, consequently impacting the rhizosphere microecological balance [27]. In research on sex determination mechanisms, Wang et al. integrated proteome and transcriptome data. By comparing mutant bisexual flowers with wild-type male flowers, they identified key regulatory genes and metabolic pathways involved in the sex conversion of *I. polycarpa*, offering molecular targets for the artificial regulation of sexual expression [28]. However, despite these valuable contributions, our understanding of fruit development characteristics across *I. polycarpa* cultivars remains incomplete. Further in-depth research is needed if we are to build a more comprehensive foundation of understanding for cultivar selection.

In light of the above-described context, in this study, we selected five provenances of *I. polycarpa* as experimental materials: SH, SG1, GG, HX, and SG2. By systematically comparing the dynamic changes in fruit growth and development among these provenances, a comprehensive analysis was conducted on the fruits’ phenotypic traits and physiological–biochemical variations. The objectives were to scientifically evaluate the quality characteristics from *I. polycarpa* from different provenances, screen superior cultivars with excellent overall performances, and finally provide substantive references and theoretical underpinnings for the genetic improvement, breeding, and cultivation of *I. polycarpa*.

## 2. Results

### 2.1. Changes in Phenotypic Traits During Fruit Growth of I. polycarpa from Different Ecotypes

From June to September, fruit phenotypic changes were observed in the different *I. polycarpa* ecotypes used in this study (Table 1 and Table 2). In terms of cluster length, the GG seed source had the longest cluster length in the M1-M4 development stages, which was about 105% higher than that of SG1, which had the shortest cluster length; SH and SG2 ecotype had middling lengths, but SG2 had longer lengths in the M1 stage, which was probably related to the rapid growth that took place in the early stage. In terms of cluster width, the differences among the four stages were small, mostly between 8 and 11 cm, but GG was significantly wider than the other ecotype in the M2-M4 stages, and the width of HX was the most stable. In terms of the cluster aspect ratio, the ratios of GG and SG2 were the highest, with the clusters being slender and long, while the ratio of SG1 was the lowest, with the clusters being thicker and shorter. In terms of the fresh weight of single clusters, the weight of GG was significantly higher than those of the other ecotypes, and the fresh weight of a single cluster of GG was higher than those of the other ecotypes. In terms of fresh weight per cluster, the weight of GG was significantly higher than those of the other ecotypes: it was 3.1 times higher than that of SH; HX had a peak at the M3 stage, but it dropped sharply by 46% at the M4 stage. In terms of the percentage of fresh weight per cluster, there were small differences between the five ecotypes in terms of the proportion of fresh weight of the fruit to the fresh weight of a single cluster; however, GG had the highest proportion at the M4 stage, indicating that it has higher economic value. In terms of the number of fruits in a single cluster, GG had the highest ratio; lower ratios were found to be associated with smaller clusters. The number of fruits for GG was much higher than those for the other ecotypes: it was 4.7 times higher than that of SG1, and the numbers of fruits in SH and SG2 were about the same in the M3 stage; however, the number of fruits in SG2 declined by 36% in the M4 stage, and the number of fruits in SH remained stable. The 1000-seed weight for SG1 was the highest among the five ecotypes, indicating good growth. The fruit shape index and the weight of single fruit showed small differences. For the fruit shape index and single-fruit weight, most fruit shape indexes ranged from 0.90 to 1.11, and fruit shapes were close to round or slightly longer; HX showed the best shape among all the ecotypes.

### 2.2. Changes in Oil and Water Content of Fruits from Different Ecotypes of I. polycarpa

Changes in oil and water content of *I. polycarpa* fruits from different ecotypes from June to September (Table 3). The water content of *I. polycarpa* fruits of different ecotypes decreased progressively with the growth stage. For example, the water content of the SG1 seed source decreased from 69% in stage M1 to 59% in stage M4, which was a significant decrease of 15%. In the same developmental stage, there were significant differences between ecotypes, and the water content of seeds from SG1 was significantly higher than those from SH and SG2 at the M1 stage, indicating that its early water metabolism was stronger. Oil content showed an increasing trend with the development stages, e.g., the oil content of seeds from GG increased from 15% at the M1 stage to 29% at the M4 stage, reflecting a significant increase of 93.3%. The differences between the ecotypes were particularly evident at the later stages. The oil content rate of seeds from GG was significantly higher than those from SG1 and HX at the M4 stage, indicating that the GG seed source was most capable of oil accumulation, followed by SH and SG2.

### 2.3. Changes of Antioxidant Enzymes in Fruit I. polycarpa from Different Ecotypes

From June to September, changes were observed in the antioxidant enzymes in *I. polycarpa* fruits from different ecotypes (Table 3). POD activity was higher in the early stages of development and decreased significantly in the later stages, showing an inverted V-shaped development trend. For example, POD activity in HX, SG1, GG, SG2, and SH decreased significantly from the M1 stage to the M4 stage, with significant decreases of 96.7%, 94.7%, 93.6%, 90.8%, and 86.7%. The POD activity of SH was 392.95 U/g at the M3 stage, which was significantly higher than that of the other ecotype. SOD activity showed an opposite trend, with SH SOD activity increasing from 238.50 U/g at the M1 stage to 337.37 U/g at the M4 stage; this was significantly higher than that of the other ecotype, with a significant increase of 41.45%. In the comparisons between the ecotypes, the SOD activity of the GG seed source was 175.13 U/g at the M2 stage, which was significantly lower than that of the other ecotype.

### 2.4. Changes in Fruit Content of I. polycarpa from Different Ecotypes

From June to September, changes were observed in the fruit content of *I. polycarpa* from different ecotypes (Table 3). Soluble sugars showed a significant decrease from the M3 stage to the M4 stage. HX showed a significant decrease from 5.86 mg·g^−1^ at the M1 stage to 2.40 mg·g^−1^, with a decrease of 58.99%, which was the sharpest decline observed among the ecotypes. This was followed by a significant decrease from 7.53 mg·g^−1^ to 3.54 mg·g^−1^ in the M1 stage in GG, with a decrease of 52.97%. SG1 and SG2 showed significant changes throughout the four stages. Soluble protein, on the other hand, showed an increasing trend, with the highest accumulation of 118.69% in the SH seed source, followed by accumulations of 90.63% and 71.95% in SG2 and GG, respectively. The M3 and M4 stages of the SG2 seed source were 25.79 mg·g^−1^ and 31.88 mg·g^−1^, respectively, which were significantly higher than those of the other ecotypes.

### 2.5. Correlation Analysis of Fruit Quality Indexes of I. polycarpa

**Figure 1:**
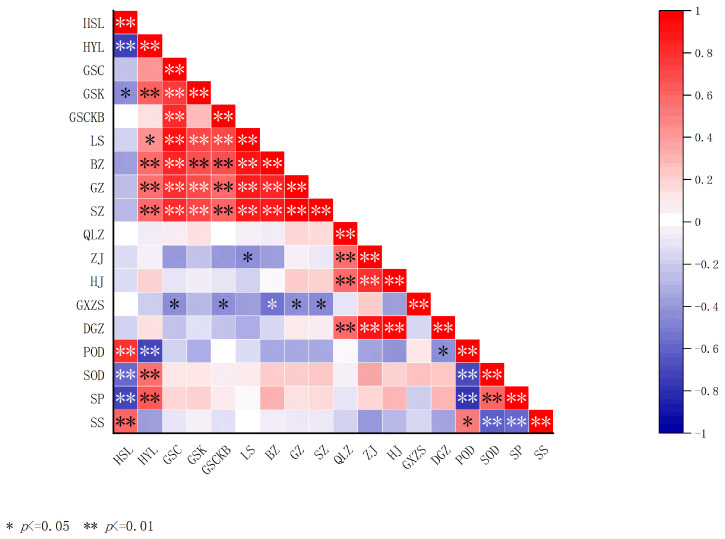
Correlation analysis of fruit quality indexes of different ecotypes *I. polycarpa*. Water content is denoted as HSL, oil content is denoted as HYL, cluster length is denoted as GSC, cluster width is denoted as GSK, cluster aspect ratio is denoted as GSCKB, grain number is denoted as LS, stalk weight is denoted as BZ, fruit weight is denoted as GZ, cluster weight is denoted as SZ, 1000-kernel weight is denoted as QLZ, longitudinal diameter is denoted as ZJ, transversal diameter is denoted as HJ, fruit shape index is denoted as GXZS, and fruit weight per fruit is denoted as GXZS. Fruit shape index is denoted as GXZS, single-fruit weight is denoted as DGZ, peroxidase is denoted as POD, superoxide dismutase is denoted as SOD, soluble protein is denoted as SP, and soluble sugar is denoted as SS (detailed data values are provided in Appendix A).

Correlations among 19 traits of *I. polycarpa* fruits were investigated using Pearson correlation analysis (Figure 1). From the results, significant positive and negative correlations can be observed among several traits. Oil content and water content (r = −0.630) showed a highly significant negative correlations, and it is possible that oil accumulation came at the expense of water consumption. In addition, oil content was significantly and positively correlated with the traits of cluster width (r = 0.481), number of grains (r = 0.401), and fresh fruit weight per cluster (r = 0.522); moreover, it is possible that fruits with high oil content generally tended to have wider clusters, more grains, and higher fresh weight per cluster. Cluster length was found to be significantly and positively correlated with cluster width (r = 0.634); both were highly and significantly positively correlated with grain number (r = 0.848, 0.678) and the fresh weight of a single stalk (r = 0.802, 0.618), suggesting that increased cluster morphology may promote seediness and biomass. However, fruit shape index was significantly and positively correlated with cluster length (r = 0.738) and significantly and negatively correlated with fruit transverse and longitudinal diameter (r = −0.404, 0.413). The thousand-grain weight of the seeds was significantly and positively correlated with the fruits’ transverse diameters and the single-fruit weight (r = 0.490, 0.512). POD activity was significantly positively correlated with water content (r = 0.654), and significantly negatively correlated with the oil content and the fresh weight of single fruit cluster (r = −0.633, −0.315); these results suggest that POD activity may increase in environments with higher water content, and its activity may be inhibited in environments with higher oil content. SOD activity showed a highly significant negative correlation with POD (r = −0.617), which could be antagonistic between POD and SOD. Soluble proteins were significantly and positively correlated with SOD (r = 0.515), suggesting a synergistic effect between the antioxidant system and proteins. Soluble sugars were significantly and positively correlated with POD activity (r = 0.490), but showed a highly significant negative correlation with SOD and soluble proteins (r = −0.563, −0.521), suggesting that sugar accumulation may be dependent on the antioxidant part of the system. Meanwhile, soluble sugar showed a significant negative correlation with fruit longitudinal diameter and single-fruit weight (r = −0.359, −0.317), suggesting that higher sugar may inhibit fruits’ longitudinal development.

### 2.6. Principal Component Analysis of I. polycarpa Fruit

A principal component analysis of the 12 indicators from five *I. polycarpa* sources was followed by a dimensionality reduction analysis. Prior to the PCA, the 12 physiological parameters (e.g., soluble sugar, SOD activity) were standardized by autoscaling to account for differences in units and magnitude. The correlation matrix was used as an input for PCA to equalize the variable contributions. The results show that four principal components were extracted, with variance contribution rates of 52.1%, 24.3%, 8.2%, and 5.0%, respectively, and the cumulative contribution rate of the four principal components was 89.72%, transforming the original 12 indicators into four composite indicators (Table 4). As can be seen from Table 5, among the four main components, there are eight indicators with large loading values (>0.8): oil content, cluster length, number of grains, fruit weight, cluster weight, shank weight, fruit shape index, soluble protein, and POD. These can be used as key indicators to evaluate the quality of *I. polycarpa* fruit. Based on the data results in Table 5, quoting the coefficients in the table, four scores can be obtained.

(1) $$F_{k}=\sum_{i=1}^{12}\omega_{ki}S_{i},$$

$F_{k}$: kth principal component. *k* = 1, 2, 3, 4.

$\omega_{ki}$: the weight coefficient of variable *S_i_* in the kth principal component (i.e., the coefficient value in the original equation).

$S_{i}$: original variables (S1–S12).

F1–F4 represent principal component 1, principal component 2, principal component 3, and principal component 4; S1–S12 represent the standardized values of the average of the 12 fruit indicators (oil content, cluster length, cluster width, number of grains, shank weight, etc., respectively); the four principal components and the corresponding variance contribution rates of the principal components are taken as the weights, to obtain the following comprehensive evaluation function:

*F* = 0.52148F1 + 0.24339F2 + 0.08221F3 + 0.05011F4, (2)

According to the comprehensive evaluation function, the comprehensive scores of the evaluation of five ecotypes of *I. polycarpa* were calculated, respectively, and the higher scores indicated that the quality of the samples was better. Based on the results of the principal component analysis, the qualities of the five *I. polycarpa* ecotypes were found to be GG, SG2, HX, SH, and SG1, in descending quality order (Table 6).

## 3. Discussion

Significant differences were observed among different ecotypes, with GG performing optimally in terms of yield and oil content, which may have a strong photosynthetic capacity and promote the accumulation of photosynthetic products. In contrast, the SOD activity and soluble protein content of HX were higher, suggesting that HX may have strong resistance to adversity and is suitable for promoting cultivation under adverse conditions. In terms of developmental stages, the fresh weight of single cluster and the oil content of fruits at stage M4 generally peaked, which was consistent with the pattern of dry matter and oil accumulation in the late stage of plant reproductive growth [29]. However, the sources from SG2 showed a high fruit shape index and fruit grain number at stage M1, which may be related to early maturity characteristics and potentially valuable for shortening the breeding time.

According to the results of the correlation analyses, oil content was significantly negatively correlated with water content, which was consistent with the competitive relationship of water metabolism during plant oil synthesis [30]. Meanwhile, oil content was positively correlated with yield traits such as cluster length and cluster width, suggesting that the better cultivars may have both high yield and high oil characteristics, which provides important guidance for the breeding of *I. polycarpa* as an oilseed crop. The significant negative correlations between POD activity, oil content, and SOD activity indicated that the antioxidant enzyme system may affect the stability of oils and fats. It has been shown that high POD activity may lead to lipid oxidation [31,32], which was confirmed by the higher oil content and lower POD activity of the seeds from GG in this study. In addition, SOD activity and soluble protein showed a significant positive correlation, indicating a possible synergistic regulation between the antioxidant system and protein synthesis, which was more evident in the HX seed source.

From the results of the principal component analysis, it can be seen that the cumulative contribution of the first four principal components reached 89.72%, which can reflect the main trait variations of *I. polycarpa*. Among them, principal component 1 mainly represented yield-related traits, which were consistent with the study of economic traits of *I. polycarpa* by Zhang Jian and Liu Ying [33,34]. Principal component 1 indicated that the yield traits dominant in the phenotypic variation of *I. polycarpa* and could be used as the core index for cultivar selection. Principal component 2 mainly represented the lipid and protein indices, which were positively correlated with oil content and soluble protein and negatively correlated with POD activity, indicating that the lipid accumulation of *I. polycarpa* might have an antagonistic relationship with antioxidant enzyme activity. This finding is consistent with those presented in the study of Jiang [31] in oilseed crops: high-oil-content cultivars may be accompanied by lower antioxidant enzyme activities to reduce the oxidative loss of oil. In contrast, the high negative correlation of POD alongside the positive correlation of soluble protein and the negative correlation of sugar content suggests a trade-off between oxidative defenses and carbon allocation. Principal component 3 mainly represents the fruit morphological characteristics. Principal component 4 showed close relations to soluble sugars, suggesting that sugar accumulation may be independent of oil accumulation and yield.

## 4. Materials and Methods

### 4.1. Overview of the Test Site

The experimental site was the Forestry Experimental Site, Science and Education Park, Henan Agricultural University, Zhengzhou City, Henan Province (113.6° E, 34.87° N; see the location map in Appendix A). The area has a typical semi-arid and semi-humid continental monsoon climate, with an average annual temperature of 14.2 °C, an average multi-year precipitation of 649.9 mm, and annual sunshine of about 2400 h. The soil is a sandy loam, with a pH value of 7.85, and a soil organic matter content of about 15 g/kg.

### 4.2. Test Material

Five ecotypes of seeds named after their origin were selected: Shanxi Hanzhong (SH), Sichuan Guangyuan1 (SG1), Guangdong Guangzhou (GG), Henan Xinyang (HX), and Sichuan Guangyuan2 (SG2), were sown at 2 m × 2 m spacing. The experimental material consisted of 5-year-old seedlings (3 for each ecotype), i.e., a total of 15 plants. On 25 June (M1), 25 July (M2), 25 August (M3), and 25 September (M4) 2024, three fruit clusters were randomly selected from the periphery of the crown of the target plants at the test site, and phenotypic and physiological indices of the fruits from each of the ecotypes in each month were determined. Three technical replications were performed for each indicator.

### 4.3. Test Methods

#### 4.3.1. Determination of Fruit Phenotypic Traits

Using a straight edge to determine the length and width of the clusters, we calculated the aspect ratio and determined the number of fruits per cluster. Then, we used an electronic balance to determine the seed weight per thousand grains, the fresh weight of the fruit stalk per cluster, the fruit weight per cluster, the single fruit fresh mass, and the fresh weight per cluster. Next, we used vernier calipers to determine the fruit width and fruit length and calculate the width–length ratios. Then, the thousand-kernel weight was obtained by randomly removing pure fruit kernels from the sample, counting 100 seeds with tweezers, weighing them and, multiplying this by 10 to obtain the thousand-kernel weight.

#### 4.3.2. Determination of Water and Oil Content of Fruit

The fruit water content (*ω_w_*) was determined as follows: 30 g of fresh fruits were weighed using a balance (*m_f_*); they were put into the oven at 120 °C to bake for 0.5 h; then, the oven temperature was adjusted to 80 °C to dry them to constant weight (*m_d_*); next, the water content was calculated using the following formula:

(3) $$\omega_{w}=\frac{m_{f}-m_{d}}{m_{f}}\times 100\%$$

The fruit oil content was determined as follows (*ω_o_*): The Soxhlet extraction method was used to determine the oil content of the dried fruits. The dried fruit sample was put into the grinder. Then, 3 g was wrapped in filter paper and loaded into the Soxhlet extraction column. The appropriate amount of petroleum ether was put into the extraction bottle and extracted in a water bath for 10 h. Next, the filter paper was taken out and placed in the oven for drying to obtain the dry mass of the sample after extraction (*m_d_*). The oil content was calculated as follows:

(4) $$\omega_{o}=\frac{3-m_{d}}{3}\times 100\%$$

#### 4.3.3. Determination of Fruit Contents and Antioxidant Enzymes

Soluble protein (SP) content was determined using the Coomassie Brilliant Blue G-250 staining method; soluble sugar (SS) content was determined using the anthrone method; peroxidase (POD) content was determined using the guaiacol method; superoxide dismutase (SOD) content was determined using the nitrogen blue tetrazolium photochemical reduction method [35]. SOD and POD indicate the amount of substrate that can be converted per minute, per gram of dry fruit weight.

SP was determined as follows: A measure of 0.5 g of the fresh sample was weighed; then, 5 mL of phosphate buffer was added. Next, following ice bath grinding, centrifugation was carried out at 4 °C 12,000× g for 15 min. Then, 100 μL of supernatant and 5 mL of Kaomas Brilliant Blue G-250 reagent were added and mixed in; this was left to stand for 10 min. The absorbance was measured at 595 nm with a spectrophotometer.

SS was determined as follows: A measure of 0.5 g of the fresh sample was added to 5 mL of distilled water; this was extracted in boiling water bath for 30 min, cooled, and then centrifuged at 8000× g at 4 °C for 10 min. Next, 0.5 mL of the supernatant was taken; then, 5 mL of anthrone reagent (0.2% anthrone in concentrated sulfuric acid solution) was added, and the sample was cooled in an ice bath after being in a boiling water bath for 10 min. The absorbance value was measured at 620 nm with a spectrophotometer.

SOD was determined by adding 1.5 mL of phosphate buffer (pH 7.8), 0.1 mL of Met solution, 0.1 mL of NBT solution, 0.1 mL of EDTA-Na_2_ solution, 0.1 mL of riboflavin solution, and 0.1 mL of enzyme solution (the control was replaced by buffer) to a test tube. The reaction was placed under 4000-lux light for 20 min (25 °C), and the reaction was terminated by removing the light. Measure the absorbance value at 560 nm with a spectrophotometer.

POD was determined by taking 50 mL of phosphate buffer and adding 28 μL of guaiacol; this was heated and stirred before cooling, adding 19 μL of 30% H_2_O_2_ to form a reaction solution. Then, 3 mL of reaction solution and 1 mL of enzyme solution were added to a cuvette. The absorbance change was immediately recorded at 470 nm on a spectrophotometer reading every 30 s for 1 min.

### 4.4. Data Processing

The ANOVA and principal component analysis were performed using IBM SPSS Statistics 22. The correlation analysis and graphing were performed using Origin 2022. The location annotation was performed using ArcGIS 10.8.

## 5. Conclusions

In summary, the GG ecotype was found to be the preferred material for high-yield cultivation, especially at the M4 stage, when harvesting is economically optimal. The HX and SG1 ecotypes have the potential to be applied in quality improvement and specialty cultivation. Depending on the developmental characteristics of the different seed ecotypes, differentiated cultivation strategies can be developed. This study clarifies the key factors influencing the yield, quality, and stress-tolerance traits of *I. polycarpa* by analyzing *I. polycarpa* from multiple perspectives; thus, the results provide a theoretical basis for cultivar selection and breeding. In the future, we can combine transcriptomics or metabolomics to further resolve the molecular regulatory mechanisms of oil content and antioxidant enzyme activities, and we can carry out cultivation experiments in different ecological zones to optimize the cultivation strategy of *I. polycarpa*.

## Figures and Tables

**Table 1:** Phenotypic traits during fruit growth of *I. polycarpa* from different ecotypes (first part).

Seed Source	Developmental Stage	Cluster Length (cm)	Cluster Width (%)	Width/Length Ratio (%)	Fresh Weight per Cluster (g)	Fresh Weight of Fruit Stalks per Cluster (g)	Fruit Weight per Cluster (g)
SH	M1	21.73 ± 1.03Db	8.10 ± 0.40Ba	2.69 ± 0.11Ba	22.46 ± 6.22Ca	2.49 ± 0.71Cb	19.97 ± 5.53Ca
	M2	27.30 ± 1.85Ba	9.87 ± 1.40Aa	2.83 ± 0.24ABa	36.47 ± 7.46Ba	4.36 ± 1.03Bab	32.11 ± 6.47Ba
	M3	26.83 ± 0.95BCa	9.20 ± 0.38Ba	2.93 ± 0.22ABa	49.35 ± 11.06Ca	5.35 ± 0.65BCab	44.01 ± 10.41Ca
	M4	29.07 ± 1.65Ba	10.20 ± 0.29ABa	2.85 ± 0.08Ba	49.82 ± 8.31Ba	5.93 ± 0.94Ba	43.89 ± 7.56Ba
SG1	M1	23.56 ± 1.20CDab	8.37 ± 0.15Ba	2.82 ± 0.17Ba	30.50 ± 0.87BCa	3.47 ± 0.20Ca	27.03 ± 0.89BCa
	M2	21.33 ± 2.14Cb	9.03 ± 0.78Aa	2.36 ± 0.09Bb	32.19 ± 5.52Ba	2.96 ± 0.64Ba	29.23 ± 4.89Ba
	M3	21.77 ± 1.91Cab	8.47 ± 0.52Ba	2.57 ± 0.13Bab	42.85 ± 15.31Ca	4.31 ± 1.70Ca	38.54 ± 13.61Ca
	M4	27.20 ± 1.21Ba	10.00 ± 0.23ABa	2.72 ± 0.06Bab	55.82 ± 3.85Ba	6.01 ± 0.57Ba	49.81 ± 3.29Ba
GG	M1	34.93 ± 0.49Ab	9.70 ± 0.10Ab	3.60 ± 0.06Aab	63.33 ± 10.08Ac	8.00 ± 1.47Ab	55.33 ± 8.63Ac
	M2	37.17 ± 1.14Ab	11.43 ± 0.35Aa	3.25 ± 0.13Ab	76.73 ± 2.45Abc	7.12 ± 1.55ABb	69.61 ± 2.02Abc
	M3	39.20 ± 2.69Aab	11.40 ± 1.04Aa	3.44 ± 0.10Ab	100.05 ± 13.70ABb	9.78 ± 1.75Ab	90.26 ± 11.96ABb
	M4	43.87 ± 0.88Aa	11.27 ± 0.92Aa	3.90 ± 0.11Aa	155.56 ± 7.70Aa	15.97 ± 0.66Aa	139.60 ± 7.23Aa
HX	M1	28.13 ± 2.28BCa	8.17 ± 0.50Ba	3.44 ± 0.09Aa	53.43 ± 12.48ABb	4.81 ± 1.05BCa	48.62 ± 11.45ABb
	M2	27.43 ± 2.45Ba	10.00 ± 0.95Aa	2.75 ± 0.04ABa	85.58 ± 21.92Aab	6.80 ± 1.86ABa	78.79 ± 20.09Aab
	M3	32.10 ± 1.31Ba	10.33 ± 1.01ABa	3.18 ± 0.39ABa	110.01 ± 14.23Aa	7.55 ± 0.57ABCa	102.46 ± 13.75Aa
	M4	29.17 ± 2.06Ba	9.07 ± 0.43Ba	3.23 ± 0.28ABa	56.77 ± 7.10Bb	4.84 ± 0.31Ba	51.94 ± 6.84Bb
SG2	M1	31.07 ± 1.76ABa	8.33 ± 0.43Ba	3.73 ± 0.03Aa	56.56 ± 4.52ABa	7.82 ± 0.88ABa	48.74 ± 3.65ABa
	M2	30.47 ± 0.23Ba	10.13 ± 0.78Aa	3.05 ± 0.25Aab	63.91 ± 7.78ABa	8.81 ± 0.95Aa	55.10 ± 6.94ABa
	M3	26.10 ± 1.81BCa	9.90 ± 0.15ABa	2.64 ± 0.22Bb	65.36 ± 4.13BCa	8.58 ± 0.75ABa	56.78 ± 3.66BCa
	M4	30.00 ± 2.67Ba	9.70 ± 0.72ABa	3.17 ± 0.51ABab	48.46 ± 7.15Ba	7.84 ± 1.68Ba	40.62 ± 5.53Ba

Note: Uppercase letters after data in the same column indicate significant differences (*α* = 0.05) across ecotypes; lowercase letters indicate significant differences (*α* = 0.05) across time. Data are presented as mean ± standard error, with error values rounded to two significant digits.

**Table 2:** Phenotypic traits during fruit growth of *I. polycarpa* from different ecotypes (part two).

Seed Source	Developmental Stage	Number of Fruits per Cluster	Seed Weight per Thousand Grains (g)	Fruit Width (mm)	Fruit Length (mm)	Fruit Shape Index	Fruit Mass (g)
SH	M1	88 ± 23Ca	0.30 ± 0.03Bab	7.28 ± 0.14Ca	7.95 ± 0.03Ba	1.09 ± 0.02Aa	0.25 ± 0.03Ba
	M2	141 ± 22BCa	0.29 ± 0.00Bb	7.23 ± 0.28Ba	8.01 ± 0.15Ba	1.11 ± 0.03Aa	0.23 ± 0.02Ba
	M3	149 ± 29BCa	0.34 ± 0.02Aab	7.93 ± 0.42Ca	8.51 ± 0.21Ba	1.08 ± 0.04Aa	0.30 ± 0.03Ba
	M4	156 ± 21Ba	0.36 ± 0.00Ba	7.89 ± 0.39Ca	8.38 ± 0.33Ba	1.06 ± 0.01Aa	0.30 ± 0.04Ba
SG1	M1	75 ± 3Ca	0.40 ± 0.02Ab	8.64 ± 0.18Ab	8.67 ± 0.06Ab	1.00 ± 0.01Ba	0.38 ± 0.01Ab
	M2	71 ± 10Ca	0.43 ± 0.02Aab	9.18 ± 0.37Aab	9.17 ± 0.30Aab	1.00 ± 0.01Ba	0.44 ± 0.05Aab
	M3	82 ± 25Ca	0.37 ± 0.02Ab	9.35 ± 0.34ABab	9.08 ± 0.27ABab	0.97 ± 0.01Ba	0.45 ± 0.04Aab
	M4	96 ± 5.5Ba	0.48 ± 0.00Aa	9.82 ± 0.03ABa	9.54 ± 0.00Aa	0.97 ± 0.00Ba	0.51 ± 0.00Aa
GG	M1	228 ± 33Ab	0.36 ± 0.00ABb	7.84 ± 0.19Bb	7.25 ± 0.11Cc	0.93 ± 0.02CDa	0.24 ± 0.02Bb
	M2	263 ± 15Ab	0.40 ± 0.00Aa	7.93 ± 0.17Bb	7.34 ± 0.11Cc	0.93 ± 0.01Ca	0.26 ± 0.02Bb
	M3	319 ± 41Ab	0.37 ± 0.01Ab	8.14 ± 0.08Cb	7.71 ± 0.05Cb	0.95 ± 0.00Ba	0.29 ± 0.01Bb
	M4	449 ± 27Aa	0.38 ± 0.01Bb	8.64 ± 0.08Ca	8.17 ± 0.12Ba	0.94 ± 0.00Ca	0.35 ± 0.02Ba
HX	M1	139 ± 34BCa	0.38 ± 0.02Aa	9.11 ± 0.23Aa	8.76 ± 0.14Ab	0.96 ± 0.01BCab	0.38 ± 0.03Ab
	M2	199 ± 48ABa	0.39 ± 0.01Aa	9.04 ± 0.20Aa	8.64 ± 0.12Ab	0.96 ± 0.01BCab	0.39 ± 0.02Ab
	M3	205 ± 33Ba	0.39 ± 0.02Aa	9.97 ± 0.11Aa	9.38 ± 0.12Aab	0.94 ± 0.00Bb	0.51 ± 0.02Aab
	M4	115 ± 26Ba	0.37 ± 0.04Ba	9.98 ± 0.52Aa	9.68 ± 0.45Aa	0.97 ± 0.01Ba	0.53 ± 0.07Aa
SG2	M1	208 ± 18ABa	0.32 ± 0.01Ba	7.89 ± 0.06Bb	7.18 ± 0.11Cb	0.91 ± 0.01Da	0.24 ± 0.01Bb
	M2	198 ± 27ABa	0.33 ± 0.02Ba	8.03 ± 0.14Bab	7.41 ± 0.07Cab	0.92 ± 0.01Ca	0.27 ± 0.01Bb
	M3	185 ± 24Ba	0.24 ± 0.03Bb	8.50 ± 0.29BCab	7.69 ± 0.27Cab	0.90 ± 0.01Ba	0.31 ± 0.04Bab
	M4	118 ± 9Bb	0.34 ± 0.02Ba	8.83 ± 0.35BCa	7.99 ± 0.29Ba	0.90 ± 0.00Da	0.38 ± 0.03Ba

Note: Uppercase letters after data in the same column indicate significant differences (*α* = 0.05) across ecotypes; lowercase letters indicate significant differences *α* = 0.05) across time. Data are presented as mean ± standard error, with the error value for the number of fruits per cluster rounded to the nearest whole number, and the error values for the remaining indicators retained to two significant figures.

**Table 3:** Indexes of oil content, water content, inclusions, and antioxidant enzymes during the growth of fruits from different ecotypes of *I. polycarpa*.

Seed Source	Developmental Stage	Water Content (%)	Oil Content (%)	POD (U/g)	SOD (U/g)	Soluble Protein (mg·g^−1^)	Soluble Protein (mg·g^−1^)
SH	M1	62.7 ± 3.5Aab	9.1 ± 0.1Bc	1605.81 ± 337.60ABa	238.50 ± 28.00ABb	5.48 ± 0.13Ba	12.16 ± 1.71Ad
	M2	63.3 ± 1.2Aa	19.4 ± 1.1BCb	1684.40 ± 82.83Aa	276.88 ± 5.65Ab	5.78 ± 0.14Ba	15.86 ± 0.433Ac
	M3	61.3 ± 0.3Aab	24.7 ± 1.1Bab	392.95 ± 42.56Ab	291.99 ± 13.25Aab	6.67 ± 0.70Aa	21.61 ± 0.64Bb
	M4	56.7 ± 0.7Bb	28.0 ± 3.7Ba	214.23 ± 5.70Ab	337.37 ± 6.50Aa	2.72 ± 0.13BCb	26.60 ± 0.04BCa
SG1	M1	68.6 ± 0.1Aa	9.3 ± 0.5Bc	2267.21 ± 69.84Aa	224.44 ± 4.64Bb	7.57 ± 0.37Ad	16.57 ± 2.50Ab
	M2	62.2 ± 1.0ABbc	16.0 ± 0.8Cb	1300.60 ± 273.35Ab	245.31 ± 15.90Bb	6.47 ± 0.10Bc	15.97 ± 1.11Ab
	M3	62.8 ± 1.5Ab	19.3 ± 0.8Cab	290.46 ± 14.30Ac	253.29 ± 20.16Aab	7.02 ± 0.17Ab	20.33 ± 0.25BCab
	M4	59.2 ± 0.8Ac	19.8 ± 1.8Ca	119.49 ± 11.11Bc	291.12 ± 6.42BCa	3.84 ± 0.17Aa	21.49 ± 0.56Da
GG	M1	66.2 ± 1.6Aa	15.5 ± 0.7Ad	1981.07 ± 47.30AaB	223.42 ± 5.05Bc	7.53 ± 0.18Aa	14.49 ± 0.69Ac
	M2	63.2 ± 0.7Aab	19.6 ± 0.7ABcC	1646.45 ± 613.70Aa	175.13 ± 8.24Dd	6.97 ± 0.41Ba	17.23 ± 0.96Abc
	M3	61.6 ± 0.2Ab	21.7 ± 0.4Cb	325.64 ± 95.32Ab	289.93 ± 6.86Ab	7.09 ± 0.23AAa	18.13 ± 1.27Cb
	M4	58.3 ± 0.8ABc	28.7 ± 0.6Ba	125.93 ± 4.28Bb	315.53 ± 5.96ABa	3.54 ± 0.02Ab	24.91 ± 0.68Ca
HX	M1	66.2 ± 0.4Aa	15.6 ± 0.1Ac	1820.46 ± 391.1ABa	279.68 ± 1.17Aa	5.86 ± 0.80Ba	17.13 ± 1.03Ad
	M2	63.3 ± 0.3Ab	23.9 ± 1.9Aab	1591.15 ± 256.69Aa	222.48 ± 7.97BCb	6.07 ± 0.54Ba	11.22 ± 0.06Bc
	M3	61.7 ± 0.2Ac	25.6 ± 0.9Ba	276.27 ± 7.02Ab	287.87 ± 12.11Aa	6.28 ± 0.29Aa	19.79 ± 0.33BCb
	M4	59.3 ± 0.7Ad	20.5 ± 0.9Cb	59.26 ± 8.55Cb	278.18 ± 7.80Ca	2.40 ± 0.22Cb	27.50 ± 0.09Ba
SG2	M1	63.5 ± 0.6Aa	15.1 ± 0.7Ac	1342.72 ± 177.45Ba	242.55 ± 6.32ABab	5.93 ± 0.10Bc	16.72 ± 0.28Ac
	M2	60.6 ± 1.0Bb	20.6 ± 1.1ABb	1433.07 ± 333.15Aa	211.56 ± 4.60Cb	9.34 ± 0.65Aa	18.51 ± 1.21Ac
	M3	61.2 ± 0.6Aab	24.0 ± 1.3Bab	278.85 ± 16.65Ab	276.53 ± 8.07Aa	7.40 ± 0.18Ab	25.79 ± 1.15Ab
	M4	58.7 ± 0.9ABb	27.3 ± 1.3Ba	123.13 ± 33.82Bb	280.24 ± 19.40Ca	2.96 ± 0.21Bd	31.88 ± 0.82Aa

Note: Uppercase letters after data in the same column indicate significant differences (*α* = 0.05) across ecotypes; lowercase letters indicate significant differences (*α* = 0.05) across time. The data are expressed as mean ± standard error, with the error values for water content and oil content retained in one significant digit; the error values for the remaining indicators are retained to two significant digits.

**Table 4:** Eigenvalues and contribution values of the principal component analysis of fruit quality indices of different *I. polycarpa* ecotypes.

Principal Components	Eigenvalues	Contribution Rate (%)	Cumulative Contribution Rate (%)
F1	6.258	52.1	52.15
F2	2.921	24.3	76.49
F3	0.987	8.2	84.71
F4	0.601	5.0	89.72
F5	0.455	3.8	93.51
F6	0.303	2.5	96.03
F7	0.191	1.6	97.63
F8	0.121	1.0	98.63
F9	0.081	0.7	99.31
F10	0.064	0.5	99.85
F11	0.018	0.2	100
F12	5.524 × 10^−5^	0	100

**Table 5:** Principal component analysis eigenvectors of fruit quality indexes of different *I. polycarpa* ecotypes.

Indicator	F1	F2	F3	F4
Oil content	0.47	0.813	0.04	0.098
Cluster length	0.924	0.048	−0.163	−0.112
Cluster width	0.787	0.317	0.065	0.311
Number of fruits per cluster	0.975	−0.005	−0.066	0.002
Fresh weight of fruit stalks per cluster	0.878	0.239	−0.284	−0.038
Fruit weight per cluster	0.931	0.172	−0.106	−0.032
Cluster weight	0.941	0.181	−0.124	−0.036
Fruit shape index	−0.361	−0.06	0.904	−0.116
POD	−0.161	−0.909	0.036	0.147
SOD	0.141	0.697	0.366	−0.452
Soluble protein	0.004	0.869	−0.267	−0.268
Soluble sugar	−0.007	−0.495	−0.122	0.779

**Table 6:** Comprehensive evaluation of the fruits of different *I. polycarpa* ecotypes.

Seed Source	F1	F2	F3	F4	F (Full)	F_abridged	Rank (Full)
SH	1.006	0.857	0.842	−0.009	0.801	0.732	4
SG1	0.701	0.419	0.335	0.223	0.496	0.467	5
GG	3.827	1.110	−0.224	0.354	2.247	2.264	1
HX	2.090	1.005	0.113	0.088	1.343	1.333	3
SG2	2.115	1.124	−0.218	0.246	1.358	1.375	2

**Figure A1:**
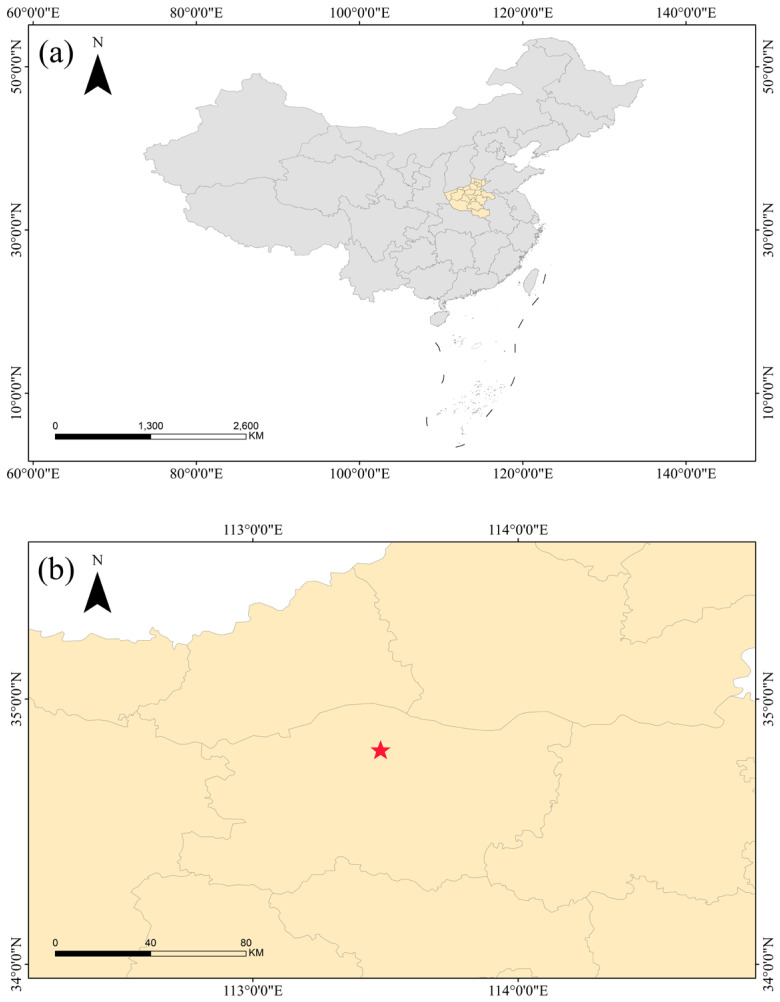
Geographical location of the test site. (**a**) The map of China and the Henan province (yellow areas) where the study was conducted. (**b**) The specific location of the test site (red star).

**Figure A2:**
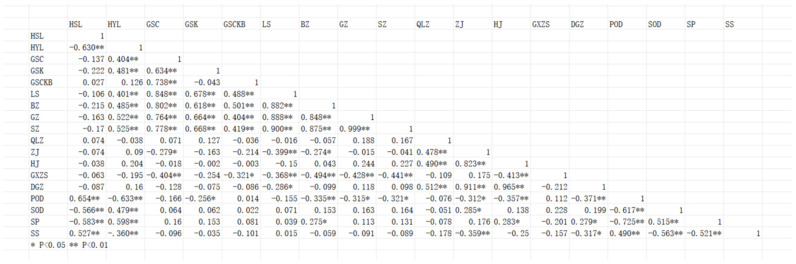
Correlation analysis of fruit quality indexes of the different ecotypes of *I. polycarpa*. Water content is denoted as HSL, oil content is denoted as HYL, cluster length is denoted as GSC, cluster width is denoted as GSK, cluster aspect ratio is denoted as GSCKB, grain number is denoted as LS, stalk weight is denoted as BZ, fruit weight is denoted as GZ, cluster weight is denoted as SZ, 1000-kernel weight is denoted as QLZ, longitudinal diameter is denoted as ZJ, transversal diameter is denoted as HJ, fruit shape index is denoted as GXZS, and fruit weight per fruit is denoted as GXZS. Fruit shape index is denoted as GXZS, single-fruit weight is denoted as DGZ, peroxidase is denoted as POD, superoxide dismutase is denoted as SOD, soluble protein is denoted as SP, soluble sugar is denoted as SS.

## Data Availability

The datasets generated and/or analyzed during the current study are available from the corresponding author upon reasonable request.
